# Application of Polyether Ketone in Oral Implantology and Prosthodontics

**DOI:** 10.7759/cureus.60175

**Published:** 2024-05-13

**Authors:** Tikeshwari Gurav, Rajiv D Bhola

**Affiliations:** 1 Department of Prosthodontics, Sharad Pawar Dental College and Hospital Dental, Datta Meghe Institute of Higher Education and Research, Wardha, IND

**Keywords:** abutment, implant, prostheses, ketone, polyether

## Abstract

Polyetheretherketone (PEEK) is a polymer that has a comprehensive range of possible uses in dental treatment. The goal of this study was to compile research findings on the substance of dental claims and highlight the upcoming predictions of PEEK in clinical dentistry. PEEK is a novel polymeric material that is yet in its preliminary stage of evolution. Biomolecules are elastic materials with remarkable mechanical strength, barrier properties, and heat resistance compared to other matrix materials. The efficacy of PEEK in clinical dentistry has been acknowledged. Polyetherketone (PEKK) and PEEK are the most commonly mentioned members of the polyaryletherketone (PAEK) family. PEEK has also found significant use in dentistry, notably in prosthodontics and implant dentistry. It also offers exceptional mechanical qualities, including high strength and toughness, making it ideal for dental implants and prostheses. It can endure the stresses of chewing and grinding, resulting in long-lasting restorations. PEEK's tooth-colored look and ability to simulate natural tooth translucency make it suitable for use in dental prostheses such as crowns and bridges. This makes it a more esthetically acceptable alternative to standard metal-based repairs.

## Introduction and background

Alloy metals such as Ti and Zr, are the most commonly utilized materials for traditional prostheses and implant restorations. To improve substructure properties, polymer materials with outstanding performance are at the leading edge of dental research, potentially decreasing the cost of prosthetic tooth rehabilitation [1]. Polymers, the most important constituents in dentistry, provide huge physical and mechanical qualities, as well as good biocompatibility. Polymers are employed in the manufacture of a diverse variety of transferable applications, restorations, and denture base materials [1]. Polyetherketoneketone (PEKK) is a novel polymer that has captured the interest of researchers because of its outstanding properties, which are suitable for diverse applications [2]. The PEKK is a greater thermoplastic polymer that is clear of methacrylate [3]. PEKK has recently gained recognition as a biomaterial with characteristics suitable for dental and medical applications [4]. It is utilized in various dental applications, encompassing therapeutic, prosthetic, and implant dentistry. It also serves multiple purposes in reconstructive, artificial, and implantable surgeries [5].

PEKK is an intriguing material for cranial and orthopedic implants. It has a wide spectrum of physiological uses due to its increased mechanical strength and the inclusion of a second ketone group. The polyaryletherketone (PAEK) family includes PEKK and polyetheretherketone (PEEK), which are its most well-known members. The PAEK family of thermoplastic polymers, utilized in engineering since the 1980s, possesses exceptional chemical and mechanical properties. These materials, however, have limitations; for instance, natural tooth attrition and bulkiness can affect the retention of prostheses and patient satisfaction. The dental industry is constantly on the lookout for improved materials that might address the shortcomings of current materials. PEEK is the most recent addition to the dental inventory, and it is touted to have superior qualities to other materials. Keeping all of this in mind, we focused this review solely on the mechanical qualities, benefits, modifications, and diverse uses of PEEK in various dental specialties.

Methodology

We conducted searches on both MEDLINE via PubMed and the Cochrane Central Register of Controlled Trials (CENTRAL) database through the Cochrane Library. We utilized various keywords: ketone, polyether, implants, abutment, and prostheses. Additionally, we examined the reference lists of potentially relevant studies to identify any additional research. Our review encompassed studies obtained from these electronic searches and relevant references found within the bibliographies of those studies.

## Review

What exactly is PEEK?

PEEK belongs to the PAEK polymer family, which has high thermal constancy (more than 300°C) and chemical and mechanical resistance. It can be a principal replacement for metallic constituents in orthopedics and trauma [6]. PEEK possesses an aromatic molecular backbone that alternates between aryl rings with ketone (-CO-) and ether (-O-) functional groups. PEEK is stable, has a low concentration (1.32 g/cm^3^), is insoluble, and has a low elastic modulus (3-4 GPa). When contrasted to Ti, PEEK offers several clinical benefits as a dental implant material. To begin with, it leads to fewer hypersensitive and allergic symptoms. According to some research, Ti can cause allergic reactions [7]. PEEK is used to fabricate various parts of the implant. The implant's body uses have been confined to bench tests, and no decision has been made concerning its placement as the implant body in the mandible. Because the automatic characteristics of PEEK and bone are more compatible, it may demonstrate inferior pressure distrustful than Ti when utilized as a dental implant body. Although PEEK can be used as a therapeutic or interim abutment, little data is known about its ultimate support. Becker (2012) demonstrated one way of determining the emergence profile in regions around dental implants using a PEEK temporary abutment. Koutouzis compared hard and soft tissue reactions to Ti and temporary PEEK abutments and found no lenient and firm tissue; there is a major difference between the two materials and responses [8].

PEKK characteristics

Mechanical and Physical Qualities

PEKK has better physicochemical characteristics than other synthetic polymers, such as glass transition and impact strength [9]. When compared with PEEK (clean and glass-reinforced), PEEK has excellent programmed properties concerning elastic deformation, stretchy, and impact strength [10]. The fatigue limit of PEKK (754 N) was found to be significantly greater than that of Zr (422 N) and Ni-Cr (586 N). The fatigue limit of PEKK compound concealed molar crowns is comparable to that of Co-Cr and polymethylmethacrylate (PMMA) veneered molar crowns (750 N). The fracturing character of PEKK was distributed among codes 1 and 2 when exposed to loads underneath the group's elastic modulus when subjected to loads less than the group's exhaustion limit, Zr and NiCr exhibited codes one and two, respectively [10]. PEKK's modulus of elasticity is comparable to that of bones, as is PEEK's. PEKK can be employed as an implant insertion biopolymer due to its strong mechanical properties and enhanced pressure distribution.

Biological Attributes

PEEK is also commonly utilized as a prosthetic and implant biocompatible material in dental care. It offers replacements that are devoid of metals, which is advantageous to people with allergies [11]. Since it provides metal-free restorations, the PEKK has good bioavailability and is considered an alternative to material and ceramics. Yuan et al. examined osteointegration in PEKK as an implant material, a measure of chemical and surface microstructure. Another acetone group in PEKK has been discovered to increase the possibility of superficial biochemical alteration. With excess ketone groups, the existence of SO_3_H on PEKK will be greater than on PEEK [12]. The addition of porosity to the surface and the introduction of HA improved the osteointegration property [12]. With more ketone groups, the occurrence of SO_3_H on PEKK will be greater than on PEEK. As a result, the surface of PEKK has a complicated surface topography, an increased surface, and a micro-rough surface, all of which impact cell activity and make it hard [13]. With excess keto groups, the presence of SO_3_H on PEEK can be higher than on PEEK. As a result, the surface of PEKK seems to have surface topography that varies, a larger surface area, and an uneven surface, all of which influence cellular activity, and the body must maintain it [14]. Walsh et al. discovered that coating PEEK with plasma-sprayed Ti enhanced the histopathological and biomechanical characteristics of the transplant contact after transplantation as compared to untreated PEEK [15].

PEEK as a Material That May Be Used in Implantation, Abutments, and Prostheses

PEEK's superior performance and isoelastic properties have prospective uses in oral implantation. PEKK offers the advantages of being tough, inexpensive, hard-wearing, and having elastic properties similar to dentin [16]. Implants for teeth made of thermoplastic resins have also demonstrated satisfactory outcomes in terms of bone contact percentages [17]. PEKK is a metal-free substance that can be used instead of a Ti implant. PEKK abutments have the advantage of being used as a framework for a fixed prosthesis and are customizable and suitable for a range of veneering materials. Combining the PEKK connection system with Ti might result in an improved quality that provides long-term implantation prosthetic stability [18]. The difficulty of posteriorly replacing missing teeth and chances for wear of acrylic teeth after some duration after implantation is addressed by a conventional complete denture (CCD) versus a fixed prosthesis of permanent full artificial teeth (implant-supported complete fixed dental prostheses (ICFDP)) [19,20]. Entire dentures (47.7%), ICFDP (19.6%), and partial dentures (20%) are the most common problems [21]. The PEKK architecture is far less traumatic on the graft and mucosa than tensile stress. As a result, PEKK construction should be limited to particular regions due to its durability. The rigid framework prosthesis provides a better stress distribution than the flexible structure prostheses. Although PEKK is commonly utilized in dental implants, it should be employed for a specific purpose, and additional study into the biochemical manipulation of PEKK to maximize abutment is needed.

Search for dental application forms

In the dentistry and medical fields, two commercial brand varieties of PEEK are primarily employed. PEEK-OPTIMA is used predominantly in the USA, whereas BioHPP is used in Europe and Asia. The two items are manufactured from modified PEEK material with improved characteristics.

PEEK-OPTIMA

Invibio Biomaterial Solutions Co. (Lancashire, UK) created PEEK-OPTIMA, the first thermoplastic implantable material, in 1999. It is a poly-aromatic semi-crystalline thermoplastic with a dissolving point of 343°C, a sintering temperature of 160°C, and a melting temperature of 145°C. PAEKs are available in three natural (unfilled) viscosity grades: high, medium, and low. According to the European Journal of Prosthodontics and Restorative Dentistry, carbon fibers enhanced properties such as strength and creep resistance. To mention a few applications, PEEK-OPTIMA is now used in dentistry as interim prosthetic abutments, healing screws, precision attachments, and implant-supported restorative frameworks. Melting and injection molding are two prominent laboratory manufacturing procedures. Computer-aided design and computer-aided manufacturing (CAD-CAM) technology may be utilized to mill frames for dentures or fixed dental prostheses (FDPs) in minutes utilizing PEEK "blanks" (Juvora, Thornton-Cleveleys, UK) [22].

BioHPP

Bredent GmbH (Germany and Sendent) created BioHPP (Bio High Performance Polymer), which is only used in dentistry treatments. The inclusion of ceramic fillers with grain widths ranging from 0.3 to 0.5 mm is included in this PEEK material modification. The manufacturer attributes the uniformity and improved polishing properties to the tiny grain size. This material is also suitable for injection molding and CAD-CAM applications. The manufacturer has certified BioHPP for three to four FDPs, telescopic restorations, and implant bar-supported prostheses that have abutments and auxiliary components [23].

PEEK Surfaces With Nanostructures

The positive impact of nanoparticles in dental applications is widely documented [24]. PEEK has also been nano-modified in recent years to increase its bioactivity and osteoconductive characteristics [24]. Particles are spread on the surface of implants using plasma torches. The plasma from the torch melts the particles, causing them to form a rough surface of the implant as they settle on it. Though spraying a bioactive layer on larger implants is appropriate, coating smaller implants is not. Due to its low melting point (about 340°C) [25], the structure of PEEK might be destroyed by excessive heat. Moreover, carbon fiber evaporation from the implant's surface because of high temperatures during the coating procedure is blamed for the low covalent attachment (2.8 MPa) of plasma-sprayed hydroxyapatite (Hap) layers on carbon-fiber-reinforced (CFR)-PEEK [26]. Dental implants are treated with osteogenic implant coatings to change their characteristics. Implants with bioactive surface coatings interact better with bone tissues, resulting in greater osseointegration [27]. Despite this, no research has been done to evaluate the apatite layer's binding strength. Because debonding of bioactive coatings might lead to complications associated with quality, examinations into the coating's quality must be done before the method involving the measurement of coated PEEK.

PEEK Nanocomposite With Bioactive Properties

Bioactive inorganic particles were introduced into PEEK by utilizing melt-blending and compression molding processes to promote bioactivity. On the other hand, incorporating bioactive HAp particles with diameters ranging from 2 to 4 m has a detrimental effect on PEEK's mechanical behavior [28]. Researchers can employ PEEK micro to make biomaterials with a variety of mechanical properties depending on the application. These bioactive nano-composites might be employed as intramedullary or further wreath replacements in addition to implants. On the other hand, incorporating bioactive HAp particles with diameters ranging from 2 to 4 m has a detrimental effect on PEEK's mechanical behavior [28]. Researchers can employ PEEK micro to make biomaterials with a variety of mechanical properties depending on the application. These bioactive nanocomposites can be used as intramedullary and additional coronal replacements in addition to implants. According to Wang et al., these restorations may have the added benefit of being anti-bacterial. However, further study is needed to discover how the composites are used and manipulated before they may be used as restorative materials [29].

Abutments for Implants Made of PEEK

PEEK may be used to make implant healing abutments with appropriate biocompatibility [30]. In a randomized, controlled clinical study, soft tissue swelling and bone resorption around PEEK and Ti abutments were not significantly different, according to Koutouzis et al. [29]. Additionally, oral infective flora attaches to PEEK abutments in the same way as Ti, Zr, and methyl methacrylate do. All of these issues are solved by a good match between the elastic modulus of bone and the PEEK layer. It protects the ribs and promotes bone remodeling. As a result, PEEK may be a viable alternative to Ti in the production of implantation abutments [29,30]. Table 1 displays the tensile adhesion capacity of PEEK.

**Table 1:** The tensile adhesion capacity of polyetheretherketone

Air gaze		Surface preparation		Sulfuric acid	
Visio-link	2.12 ± 0.78	System of adhesion stretchable bond asset (MPa)		Signum bond	1.88 ± 0.95
Signum bond	2.97 ± 0.92	Visio-link	2.006 ± 0.80	Ambarino-P60	2.18 ± 0.99

PEEK as a Material for Detachable Prostheses

PEEK, computer-aided design, and computer-aided trade technologies can be used to create dentures. Tannous et al. hypothesized that PEEK denture clasps had smaller retaining pressures than Co-Cr straps [31]. However, because the study was done on an artificial metallic crown, it is uncertain how effective the appealing PEEK buckles would be in retaining prostheses in a clinical setting. PEEK is also being used in the development of a detachable obturator [32]. However, more studies are required to evaluate the efficacy of PEEK obturators in traditional acrylic prostheses. So far, no medical trials or review articles have been published on the use of PEEK prostheses. However, considering PEEK's exceptional mechanical and biological properties, it is not astonishing that dentures manufactured from the polymer will become prevalent mostly in the near term.

PEEK-Based Abutments

Several ways of treating the face of PEEK to allow adhesion to composite resin veneers have been presented. Even though wind exfoliation with silicon covering results in a more soluble aqueous surface, H_2_SO_4_ results in a harsh and modified shallow that enables it to link more effectively with hydrophilic composite resins (maximum shear strength: 19.0-3.4 MPa) [33]. After 28 days at 37°C in water, H2SO4 etching for 60-90 seconds produced shear bond strengths to resin composite cement of 15.3, 7.2, and 9.3 MPa. There were no substantial differences in the tensile interfacial adhesion of PEEK restorations and abutments of dentin after air attrition or H_2_SO_4_ washing [34,35].

CAD-CAM Machined PEEK Permanent Prosthesis

CAD-CAM manufactured materials and PMMA permanent prostheses outperform standard fixed dentures in terms of mechanical properties [36,37]. PEEK is yet another substance that may be used to replace PMMA in CAD-CAM restorations. It has been proposed that PEEK permanent prostheses created using CAD-CAM dentures are more fracture-tough than compacted sandy or bead PEEK dentures [38,39]. No scientific investigations, however, have been conducted to compare the abrasion produced by PEEK peaks on teeth to that generated by other components such as metals and ceramics. As a result, it is currently unclear whether PEEK crowns can work effectively with dentin and enamel. A PEEK fixed partial denture with high abrasion resistance and mechanical capabilities, as well as the previously indicated suitable binding with materials or dentition, must have a longer life [40,41].

## Conclusions

Due to its strength and modulus, which are close to dentin and bone, PEEK may be used for a range of dental prostheses and implant placement. The mechanical properties of PEEK dental implants get hampered when trying to increase their bioactivity. PEEK can be used to produce CAD-CAM fixed and other prostheses because of its greater strength than acrylic. More research and clinical trials are needed for a better understanding of PEEK and its potential for future application in dentistry. PEEK materials offer the right physical, motorized, and chemical properties for application in dentistry, including restorative materials, immovable prosthetic devices, and dental biomaterial implants, to name a few. Modifications and improvements to material properties may lead to greater medical applications. Long-term assessments are necessary because PEEK was finally confirmed.
